# Supplementary material for: The Systems Biology Research Tool: evolvable open-source software
Source: BMC Syst Biol. 2008 Jun 29;2:55. doi: 10.1186/1752-0509-2-55 (PMC2446383; doi:10.1186/1752-0509-2-55)
Supplement: Additional file 1 — SBRT Archive. An archive of the current version of the Systems Biology Research Tool. [file 1752-0509-2-55-S1.zip › sbrt-1.4.0/doc/developers_guide/api/sbrt/kernel/class-use/KernelProcess.html]

Uses of Interface sbrt.kernel.KernelProcess


|  |  |  |
| --- | --- | --- |
| **Overview** \| **Package** \| **Class** \| **Use** \| **Tree** \| **Deprecated** \| **Index** \| **Help** | |  |
| PREV   NEXT | **FRAMES**    **NO FRAMES**     **All Classes** |  |


---


## **Uses of Interface sbrt.kernel.KernelProcess**

| Packages that use KernelProcess | |
| --- | --- |
| **sbrt.kernel** | Provides interfaces to define processes. |
| **sbrt.kernel.fba.proc** | Provides classes and interfaces for executing Flux Balance Analysis processes. |
| **sbrt.kernel.math.algebra.proc** | Provides classes and interfaces for executing algebra-related processes. |
| **sbrt.kernel.math.comb.proc** | Provides classes and interfaces for performing combinatorial analysis-related processes. |
| **sbrt.kernel.math.geom.proc** | Provides classes and interfaces for performing geometry-related processes. |
| **sbrt.kernel.math.graph\_theory.proc** | Provides classes and interfaces for performing graph theoretical processes. |
| **sbrt.kernel.math.stat.proc** | Provides classes and interfaces for performing statistical processes. |
| **sbrt.kernel.utilities.proc** | Provides classes and interfaces for performing utility processes. |
| **sbrt.shell.mng** | Provides classes and interfaces for creating process managers. |

Uses of KernelProcess in sbrt.kernel

| Subinterfaces of KernelProcess in sbrt.kernel | |
| --- | --- |
| `interface` | `MonitoredProcess<R>`             This interface is used to represent monitored processes. |

Uses of KernelProcess in sbrt.kernel.fba.proc

| Classes in sbrt.kernel.fba.proc that implement KernelProcess | |
| --- | --- |
| `class` | `CatalystDeletionProcess`             This class is used to perform catalyst deletion experiments. |
| `class` | `CdHarFluxVectorProcess`             This class is used to perform coordinate direction hit-and-run flux vector experiments. |
| `class` | `CvCatalystDeletionProcess`             This class is used to perform constraint-variation catalyst deletion experiments. |
| `class` | `CvFbaOptimizationExperiment`             This class is used to perform constraint-variation FBA optimization experiments. |
| `class` | `CvFbaOptProcess<R>`             This class is used to represent constraint-variation Flux Balance Analysis experiments. |
| `class` | `CvFluxIntervalProcess`             This class is used to perform constraint variation flux interval experiments. |
| `class` | `CvObjectiveFunctionProcess`             This class is used to perform constraint-variation objective function experiments. |
| `class` | `CvRxnDeletionProcess`             This class is used to perform constraint-variation reaction deletion experiments. |
| `class` | `CycleIdProcess`             This class is used to execute steady state cycle identification processes. |
| `class` | `ExtremeCurrentProcess`             This class is used to perform extreme current experiments. |
| `class` | `FbaOptimizationProcess`             This class is used to perform basic Flux Balance Analysis optimization. |
| `class` | `FbaProcess<R>`             This abstract class is used to represent Flux Balance Analysis experiments. |
| `class` | `FbaSolverProcess`             This class is used to solve Sv = 0. |
| `class` | `FluxActivityProcess`             This class is used to perform flux activity experiments. |
| `class` | `FluxCapProcess`             This class is used to perform a flux cap identification. |
| `class` | `FluxIntervalProcess`             This class is used to perform flux interval experiments. |
| `class` | `FluxomeReducerProcess<T extends Fluxome>`             This class is used to execute fluxome reduction processes. |
| `class` | `FluxPlasticityProcess`             This class is used to perform flux plasticity experiments. |
| `class` | `InitialPointGenerator`             This class is used to perform initial point experiments. |
| `class` | `NetworkInfoProcess`             This class is used to gather basic information about a fluxome. |
| `class` | `ObjectiveFunctionAnalysis`             This class is used to perform objective function experiments. |
| `class` | `RandomConstraintsGeneratorV1`             This class is used to generate random flux constraints. |
| `class` | `RandomEnvGeneratorV1`             This class is used to generate random environments for use in flux balance analysis. |
| `class` | `RandomObjectiveGenerator`             This class is used to generate random objective functions. |
| `class` | `RxnDeletionProcess`             This class is used to perform reaction deletion experiments. |
| `class` | `StoichVariationProcess`             This class is used to perform stoichiometry-variation FBA optimizations. |
| `class` | `SvCdHarFluxVectorProcess`             This class is used to perform space variation coordinate direction hit-and-run flux vector experiments. |
| `class` | `SvInitialPointProcess`             This class is used to perform constraint variation initial point experiments. |

Uses of KernelProcess in sbrt.kernel.math.algebra.proc

| Classes in sbrt.kernel.math.algebra.proc that implement KernelProcess | |
| --- | --- |
| `class` | `LinearSolverProcess<V>`             This class is used to solve systems of linear equations. |
| `class` | `VectorIntersection<K,V>`             This class is used to intersect vectors whose variables (that is, map keys) are not identical. |

Uses of KernelProcess in sbrt.kernel.math.comb.proc

| Classes in sbrt.kernel.math.comb.proc that implement KernelProcess | |
| --- | --- |
| `class` | `SingleElementUnionProc<E>`             This class is used to wrap the single element union computer as a process. |
| `class` | `StrictSingleElementUnionProc<E>`             This class is used to wrap the strict single-element union computer as a process. |

Uses of KernelProcess in sbrt.kernel.math.geom.proc

| Classes in sbrt.kernel.math.geom.proc that implement KernelProcess | |
| --- | --- |
| `class` | `CdHarProcess<V>`             This class is used to generate random, uniformly distributed points from the interior of a convex polytope. |

Uses of KernelProcess in sbrt.kernel.math.graph\_theory.proc

| Classes in sbrt.kernel.math.graph\_theory.proc that implement KernelProcess | |
| --- | --- |
| `class` | `PathIdProcess<N>`             This class is used to identify all the simple paths in a directed graph. |

Uses of KernelProcess in sbrt.kernel.math.stat.proc

| Classes in sbrt.kernel.math.stat.proc that implement KernelProcess | |
| --- | --- |
| `class` | `CorrelationAnalysis<V,T extends CorrResult>`             This class is used to perform correlation experiments. |
| `class` | `MultipleCorrelationAnalysis<V,T extends CorrResult>`             This class is used to perform multiple correlation analyses. |
| `class` | `MwUAnalysis<V>`             This class is used to perform Mann-Whitney U tests. |

Uses of KernelProcess in sbrt.kernel.utilities.proc

| Classes in sbrt.kernel.utilities.proc that implement KernelProcess | |
| --- | --- |
| `class` | `IntervalComparison<V>`             This class is used to compare sets of intervals. |
| `class` | `VariableParticipation<E extends MathExpr<V>,V>`             This class is used to organize a collection of mathematical expressions according to the variables they contain. |
| `class` | `VectorComparison<V>`             This class is used to compare sets of intervals. |

Uses of KernelProcess in sbrt.shell.mng

| Methods in sbrt.shell.mng that return KernelProcess | |
| --- | --- |
| `KernelProcess<?>` | `KernelProcessManager.getProcess()`             Returns the process this process manager controls. |

---


|  |  |  |
| --- | --- | --- |
| **Overview** \| **Package** \| **Class** \| **Use** \| **Tree** \| **Deprecated** \| **Index** \| **Help** | |  |
| PREV   NEXT | **FRAMES**    **NO FRAMES**     **All Classes** |  |


---
